# Microstimulation of Frontal Cortex Can Reorder a Remembered Spatial Sequence

**DOI:** 10.1371/journal.pbio.0040134

**Published:** 2006-04-25

**Authors:** Mark H Histed, Earl K Miller

**Affiliations:** **1**The Picower Institute for Learning and Memory, RIKEN-MIT Neuroscience Research Center, and Department of Brain and Cognitive Sciences, Massachusetts Institute of Technology, Cambridge, Massachusetts, United States of America; National Institute of Mental HealthUnited States of America

## Abstract

Complex goal-directed behaviors extend over time and thus depend on the ability to serially order memories and assemble compound, temporally coordinated movements. Theories of sequential processing range from simple associative chaining to hierarchical models in which order is encoded explicitly and separately from sequence components. To examine how short-term memory and planning for sequences might be coded, we used microstimulation to perturb neural activity in the supplementary eye field (SEF) while animals held a sequence of two cued locations in memory over a short delay. We found that stimulation affected the order in which animals saccaded to the locations, but not the memory for which locations were cued. These results imply that memory for sequential order can be dissociated from that of its components. Furthermore, stimulation of the SEF appeared to bias sequence endpoints to converge toward a location in contralateral space, suggesting that this area encodes sequences in terms of their endpoints rather than their individual components.

## Abstract 2

Errors induced by microstimulation of the supplementary eye fields while monkeys performed a remembered series of saccadic eye movements give insight into the nature of sequence coding in this cortical area.

## Introduction

Understanding how the brain represents sequence information is key to understanding complex, goal-directed behavior. Many theories of sequential behaviors posit “associative chaining” codes [ 1, 2], where neurons representing earlier sequence elements activate neurons representing later elements. In these models, the order of actions in a behavioral sequence is represented by the order of neurons' firing; specific neurons fire at specific points in the sequence [ 3, 4]. Evidence for this type of coding has been found during execution of action sequences such as birds singing remembered songs [ 5] and rats running through a maze in a fixed sequence [ 6– 8]. Associative chaining-like activity has also been found in the primate frontal cortex, which contains areas that both human and monkey studies indicate are involved in sequencing [ 9– 11]. The monkey supplementary eye field (SEF) contains neurons that fire selectively for a given eye movement and its ordinal position during execution of a learned sequence [ 12, 13]. Likewise, neurons in the medial supplementary motor area are selectively activated during sequences of arm movements [ 14].

Thus, a “chain-like” type of sequence encoding seems common during action execution, but what about planning a movement sequence? Models of planning behavioral sequences suggest a hierarchical coding scheme that includes higher-level representations [ 15, 16] of the entire movement that are held in memory before the movement starts. The key element of these schemes is that the coding for higher-level quantities, like movement goals, is separate from the coding for individual movements or motor details. To differentiate between different types of sequence coding schemes, we examined the SEF, an oculomotor area in the frontal cortex linked to the volitional control of eye movements [ 17– 22].

The SEF and another frontal lobe oculomotor area, the frontal eye field (FEF) both contain neurons that are sequence-selective [ 11– 13, 23], though sequence tuning is stronger in the SEF than the FEF [ 24]. These neurons show activity that reflects not only the next upcoming movement but also future movements in a learned sequence. However, because single neurons have been shown to often reflect both the exact movements and their sequential order (e.g., Figure 4 and 5 in [ 12], Figure 3 in [ 13]), physiological studies have not resolved the question of whether there is a higher-order representation of sequence that can be dissociated from the movement details. Here, we use microstimulation to address this question. We show that microstimulation of the SEF during a memory delay affects the order of saccades to two remembered locations, but does not affect either the monkeys' ability to saccade to the locations that were cued or saccade parameters like accuracy, velocity, or latency. By contrast, microstimulation of the FEF had a much weaker effect.

## Results

**Figure 1:**
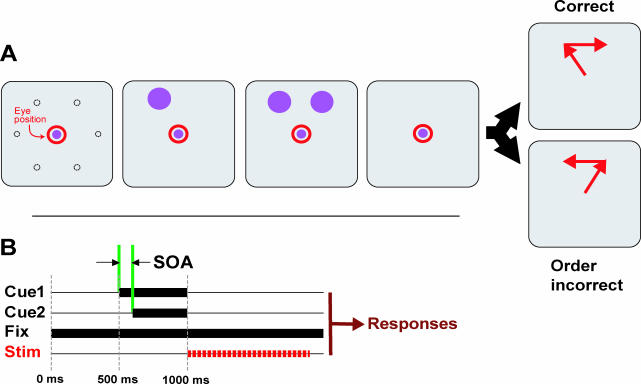
Sequential Memory Task (A) The animals fixated a central spot for 500 ms, then the two cues appeared, separated by a short interval (the SOA). The cues were extinguished together, and the animals maintained fixation over a 1-s memory delay. To respond correctly, they then saccaded to the remembered locations of the cues in the order they appeared. Dotted circles represent possible cue locations (not displayed to animal B, visible to animal A; see Materials and Methods); cues were always presented at adjacent positions. (B) Timing of trial events. Intracortical microstimulation was applied for the first 900 ms of the 1,000-ms delay period.

**Figure 2:**
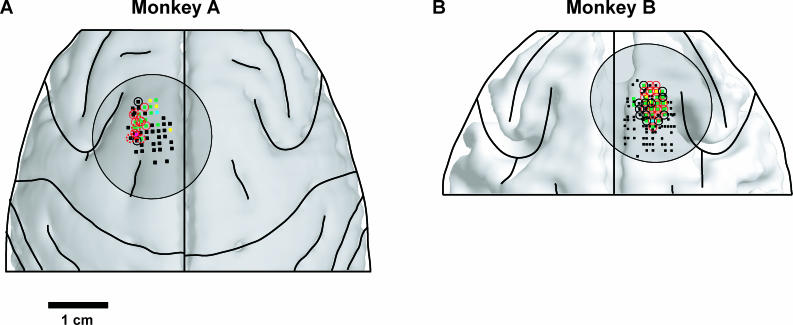
Stimulation Locations in SEF Stimulation sites are plotted relative to brain anatomy constructed from MRI images. (A) Sites in monkey A; MRI image shows the surface of the gray matter. (B) Monkey B; MRI image shows the outer surface of the white matter, i.e., the border between the gray and white matter. (This was the most salient boundary for this MRI; see Materials and Methods.) Black lines show locations of sulci, circles are locations of recording chambers. Squares, result of suprathreshold mapping stimulation (threshold less than or equal to 50 μA). Green squares show convergent saccades elicited; yellow, vector saccades; cyan, stimulation produced fixation; magenta, pursuit movements elicited; black, no effect. Red circles, stimulation caused response order bias for at least one cue pair. Black circles, no significant order effect.

We trained monkeys to perform a sequence of eye movements from memory ( Figure 1). On each trial, two (of six) locations were cued randomly, usually in order but sometimes simultaneously (see below). Then, after a short delay, monkeys were required to saccade to those locations in the order in which they were cued. We microstimulated SEF sites during the memory delay, when monkeys were holding sequence information in short-term memory and planning the saccades. We systematically varied the interval between the cues' onsets (the stimulus onset asynchrony [SOA]) to manipulate the difficulty of detecting their order. Microstimulation, below threshold for eliciting eye movements, was applied to the SEF during the 1-s memory delay ( Figure 2; see Materials and Methods).

**Figure 3:**
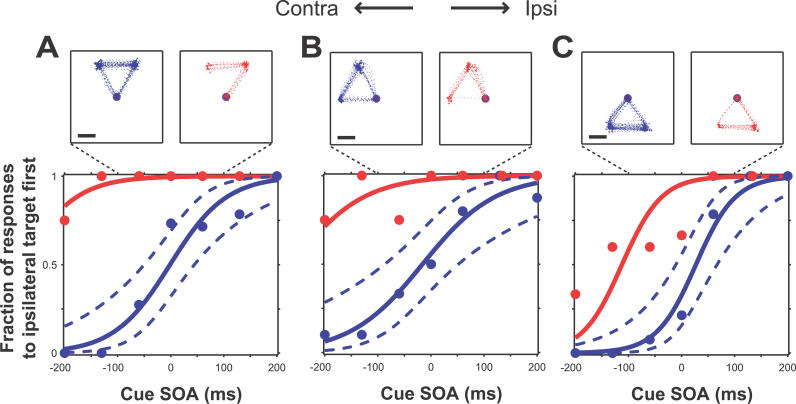
Effect of Stimulation at Three SEF Sites Psychometric behavioral curves are shown in the lower panels. Horizontal axis, SOA; vertical axis, fraction of trials where the first saccade was directed to the more ipsilateral target. Blue, unstimulated trials; red, stimulated trials. Solid lines, logistic regression curve. Dotted blue lines, simultaneous 95% confidence interval. Number of trials for each plot (stim/unstim): (A) 28/96; (B) 30/67; (C) 28/95. Upper panels: eye traces from every trial of the three central SOAs: 0, −60, and +60. Scale bar: 5°; fixation spots not drawn to scale. In each case stimulation strongly biased the animal to choose the more ipsilateral target first (and thus the more contralateral second).

**Figure 4:**
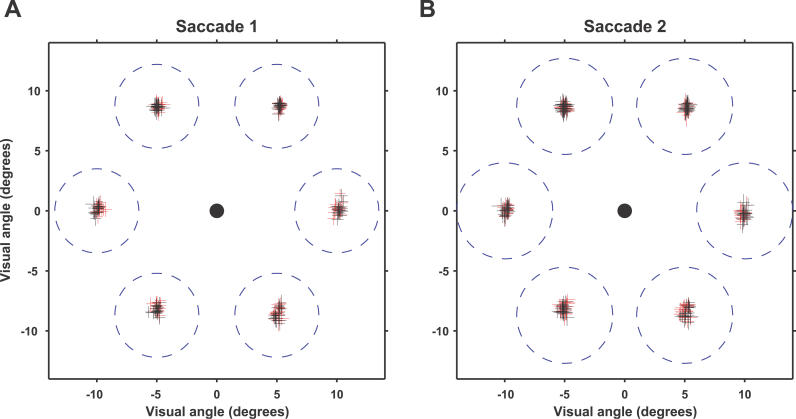
Saccade Endpoint Locations Are Unchanged by Stimulation Endpoints of first saccade (A). Endpoints of second saccade (B). (Data from animal B only; for this animal, no visual cues were available to possibly guide saccades.) Plus signs indicate mean (intersection point) and standard deviations in x and y directions (length of horizontal and vertical bar in plus symbol) of saccade endpoints over an experimental session. Stimulated endpoints are plotted in red, unstimulated endpoints in black. Blue dashed circles indicate the size of the window outside which a saccade endpoint would be considered incorrect.

**Figure 5:**
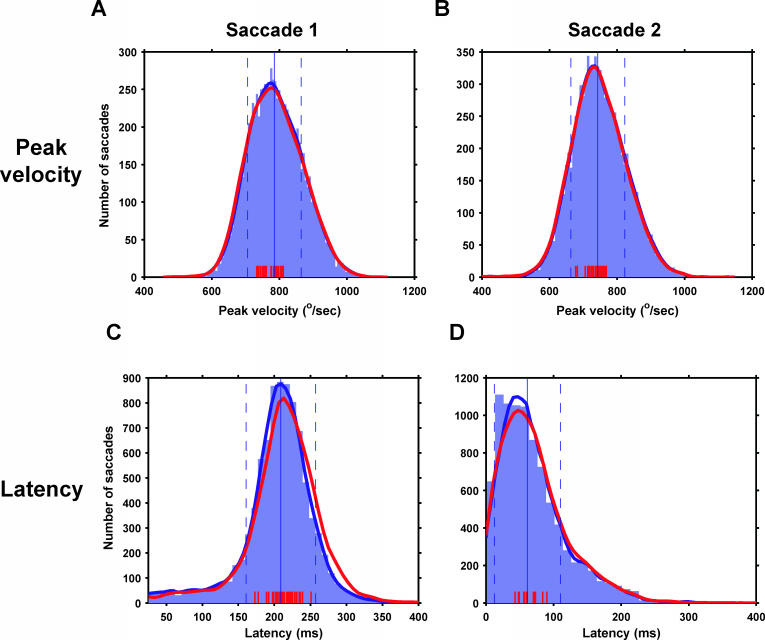
Velocities and Latencies Are Unchanged by Stimulation Peak velocities of first and second saccades, respectively (A and B). Blue region, histogram of peak saccade velocities on unstimulated trials. Thick blue lines (largely hidden by red lines), kernel density estimate of this distribution. Thick red lines, estimate of the distribution of *stimulated* peak velocities. (Histogram not shown.) Solid and dashed blue vertical lines, mean and s.d. of unstimulated distribution. Red tick marks (bottom), the mean peak velocity for stimulated trials for a single stimulation site. Note that all red tick marks lie within 1 s.d. of the mean of the unstimulated distribution. Latencies of first and second saccades (C and D) use the same conventions.

If SEF delay activity played a major role in remembering the individual cued locations, we would expect that microstimulation might sometimes produce target errors (a saccade to a “wrong” location, i.e., one that was not cued). If it played a role in representing sequence information, we might sometimes see sequence errors (saccades to the cued locations but in the wrong order). Furthermore, if the SEF represented all of the sequence components, we would sometimes produce errors in making the first saccade and sometimes in making the second saccade, as if we were randomly selecting different elements of the sequence chain. We found a consistent pattern of results. SEF stimulation produced sequence errors, not target errors, and it did so in a systematic way: SEF stimulation biased the sequential movements so that the endpoints seemed to converge toward a zone in the contralateral hemifield.

The effect of microstimulation at three example SEF sites is shown in Figure 3. The lower panels show psychophysical curves representing the proportion of trials in which the monkey picked the ipsilateral target first as a function of the SOA (unstimulated trials are plotted in blue and stimulated trials in red). Positive SOAs indicate that the more ipsilateral target appeared first, and negative SOAs, the more contralateral target first. At zero SOA, the targets appeared simultaneously; the animals “guessed” at the correct order and were rewarded randomly on 50% of trials. At all three sites ( Figure 3A– 3C), stimulation biased the monkeys to choose the ipsilateral target first and the contralateral second, leading to a significant upward shift in the psychophysical curve on stimulated trials, shown in red ( *p* < 10 ^−4^, logistic regression [ 25, 26]). Across the two animals, stimulation at 25 of 55 sites (45%) produced a significant bias (at *p* < 0.01, corrected for multiple comparisons), while control performance was unaffected. The bias is also apparent in the eye traces for the three smallest SOAs (−60, 0, +60 ms; Figure 3, upper panels). While stimulation produced a bias of the curve, making the animals more likely to choose one of the two possible sequences, it did not degrade the animals' ability to discriminate order ( *p* = 0.22, binomial test; see Materials and Methods). If stimulation affected discrimination, it would have changed the slope of the curve, as the ability to perform both sequences would be affected. Instead, stimulation caused animals to perform one sequence more often than the other, resulting in a bias.

More important, while SEF stimulation can evoke single saccades that are contraversive to the stimulated hemisphere [ 27– 29], we found that the first saccade was *ipsiversive* on most stimulated trials when one cue was contra- and the other ipsilateral (e.g., Figure 3A and 3C), resulting in a contraversive endpoint. In principle, this initial ipsiversive saccade might be explained by a release from the monkeys resisting the contraversive bias during delay epoch stimulation (as they maintained central fixation). However, this did not seem to be the case. First, there was no discernable effect of stimulation on the monkeys' pattern of microsaccades during fixation. Second, and more important, stimulation seemed to affect sequencing per se: an effect on just one of the two saccades would have produced a different pattern of errors, leading to many target errors (saccades to a location that was not cued). In fact, stimulation did not disrupt the monkeys' ability to saccade to those locations that were cued, only their correct *order.* Monkeys made target errors on fewer than 2% of trials, and this was unaffected by stimulation.

Saccade metrics and dynamics were also not affected by stimulation, providing further evidence that stimulation does not affect the components of the sequence. Figure 4 shows the endpoint of the first ( Figure 4A) and second saccade ( Figure 4B) on stimulated (red) and unstimulated (black) trials. There was no significant difference. Peak velocity ( Figure 5A and 5B) and saccade latencies ( Figure 5C and 5D) were also not affected by stimulation.

**Figure 6:**
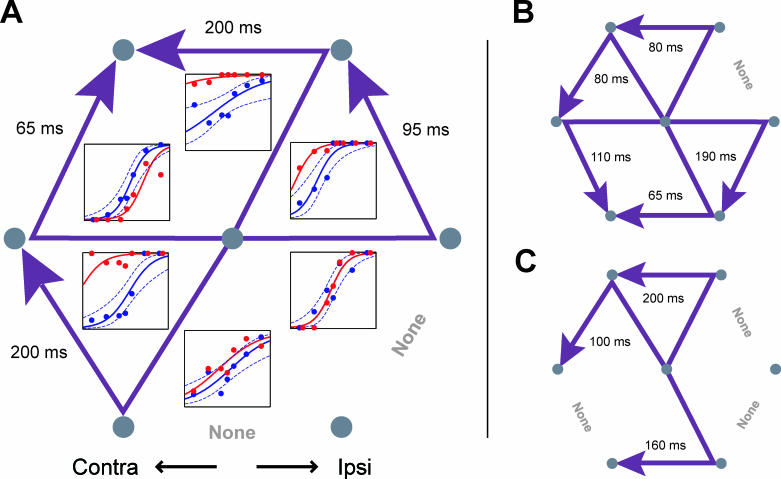
Stimulation Biases Endpoints toward a Contralateral Zone The pattern of stimulation-induced bias at all pair locations from three different SEF sites (A–C). Purple arrows, the sequence that was preferred on stimulation trials. Numbers outside each pair, magnitude of the shifts (milliseconds of SOA). Psychometric curves (conventions as in Figure 2) are shown for each pair in (A).

**Figure 7:**
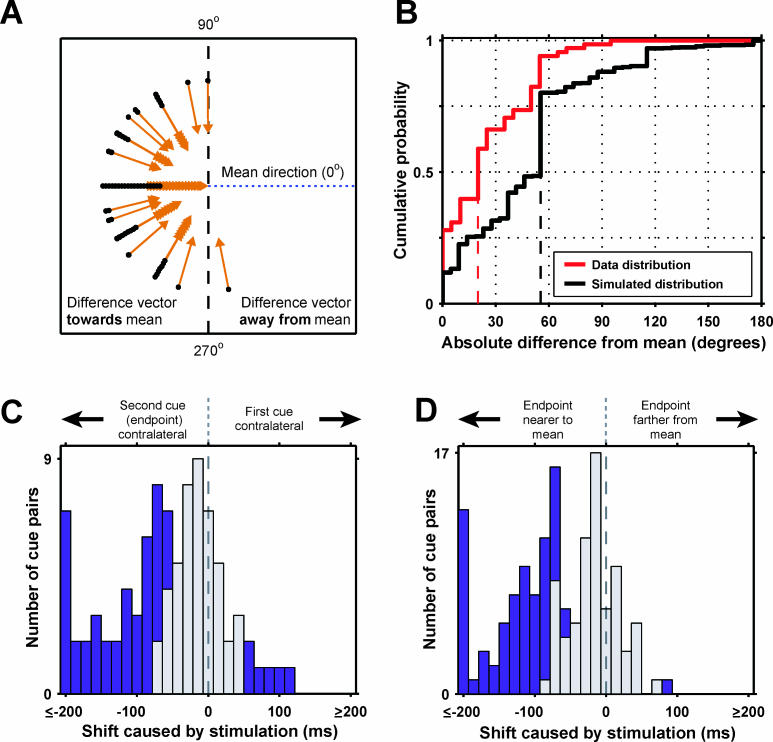
Bias Directions Are Convergent (A) Difference vectors around each site's mean vector. Black dots, origin of each difference vector; vectors are offset radially, so all are visible. Vector origins beyond the 90–270° vertical line (1/59; 23 expected by chance) indicate a shift direction *away* from the mean. (B) Cumulative distribution of absolute differences between each vector and the site's mean (summary of the effect in [A]). Red line, true distribution; black line, simulated null distribution; dashed vertical lines, medians. The distributions are significantly different ( *p* < 10 ^−6^; Kolmogorov-Smirnov statistic = 0.36). (C and D) Magnitude of sequence effects. (C) Histogram of biases for pairs with one target ipsilateral and one contralateral to fixation. Horizontal axis, size of shift. Purple, statistically significant shifts ( *p* < 0.05); gray, non-significant shifts. Positive shifts signify that the contralateral target was chosen *first,* negative shifts that the contralateral target was chosen *second.* (D) Histogram of bias directions relative to difference vector mean direction, conventions as in (C).

The effect of stimulation on the whole sequence, and not just one of the individual saccades, was also supported by observations that it seemed to bias the final endpoints of the sequences to converge to a zone in contralateral space. Figure 6 shows which sequences were significantly biased by stimulation for three different SEF sites (at a threshold of p < 0.05, logistic regression, corrected for multiple comparisons). For example, in Figure 7A, the arrow showing a first saccade up and to the right and a second saccade to the left (i.e., first to “one o'clock” and then to “11 o'clock”) indicates that stimulation resulted in a significantly greater tendency to make that sequence over the alternative from that target pair (to 11 o'clock, then to one o'clock). The magnitude of the bias is expressed as the shift in milliseconds of SOA of the psychometric function, i.e., the change in SOA needed to produce the same behavioral effect (biases with absolute value greater than or equal to 200 ms were set to 200 ms). When one cue was contralateral and one ipsilateral, stimulation caused a bias toward the contralateral endpoint ( Figures 3A, 3B, and 6A). But when both cues appeared on one side, the biases were not explained merely by a final saccade to the most contralateral point. Sometimes, the bias could be for a final saccade directed in the ipsilateral direction (e.g., in Figure 7A, the second saccade from nine o'clock to 11 o'clock). Thus, SEF stimulation did not simply bias the final saccade contraversively, but instead biased the endpoints of the sequences to converge to a zone in the contralateral hemifield. For example, the sequences in Figure 6A seem to converge toward a zone in the upper contralateral field; in Figure 6B, they converge toward a zone in the lower contralateral field. Note that if stimulation were affecting the first saccade alone (either directly or by “releasing” resistance to a contralateral bias during the memory delay), we might expect divergence of the saccade endpoints, not convergence. For example, for the site shown in Figure 6B, examine two pairs of targets: first, the pair directly below fixation (labeled in Figure 6B by its 65-ms shift), and second, the pair below and left of fixation (shift of 110 ms). Stimulation biases the endpoints of the two sequences to converge to a point in the lower contralateral hemifield. If stimulation acted on the first saccade, it could explain one of the bias directions, but not both. Assume that the pair below fixation (65-ms shift) had its first saccade biased toward five o'clock, or 300°, to explain the direction of bias that we observed. Then the other pair (110-ms shift) would have its first saccade biased to seven o'clock (240°, the first saccade that was nearest to 300°), leading to a sequence order opposite to what was observed. In sum, any explanation of these data in terms of effects on the first or second saccade alone would not explain the coherent pattern of convergent endpoints that we observed or the monkeys' very low rate of target errors.

The convergent nature of the biases is summarized statistically in Figure 7A and 7B. For each site with two or more target pairs that showed a significant effect of stimulation, we calculated the vector difference between the biased and non-biased endpoint. The mean difference vector for each SEF site points toward the approximate final direction or zone to which stimulation biases the endpoint of the sequences. For example, in Figure 6A, the mean difference vector would point up and to the left. If the difference vector for one pair of cues points in this direction (i.e., within a 180° interval centered on the mean vector), it indicates the bias was convergent. The difference vector also pointed in the same direction as the vector of the second saccade (measured from the position of the eye after the first saccade), but the lack of target errors make it unlikely that stimulation affected the second saccade in this vectorial way. Figure 7A plots all the difference vectors as a function of distance from their mean vector. Almost all of the difference vectors are toward the mean vector for each site (between 90–270°), indicating that endpoints converge to a similar final direction. Only one vector was in a different direction (compared to 23 that would be expected by chance; p < 10 ^−10^, χ ^2^ test). Since the direction of each vector affects the direction of the site's mean vector (because the mean is taken over all pairs at a single site), we confirmed the convergence using simulation. We calculated the null distribution of the absolute difference in angle between each difference vector and the mean vector at that site, assuming the difference vector directions (the bias directions caused by stimulation) were independent ( Figure 7B; see Materials and Methods). The distributions were significantly different (p < 10 ^−6^, Kolmogorov-Smirnov test), confirming that endpoints selected by stimulation were clustered around the region given by the mean vector.

The magnitude of the biases caused by stimulation is illustrated in Figure 7C and 7D. Statistically significant shifts (p < 0.05, corrected for multiple comparisons) are purple and non-significant shifts are gray. Figure 7C shows all cue pairs where one cue was ipsilateral to the fixation point and the other cue contralateral. The distribution shows a strong leftward shift, indicating a bias toward sequences with contralateral endpoints. As noted above, stimulation causes a bias away from contraversive first saccades. Figure 7D shows the same plot (incorporating all cue pairs) where a mean difference vector could be calculated, not just the pairs where one cue is ipsi- and the other contralateral. This distribution is similarly strongly biased to the left (p < 10 ^−6^, Wilcoxon test), showing that stimulation causes convergence around the difference vector mean.

**Figure 8:**
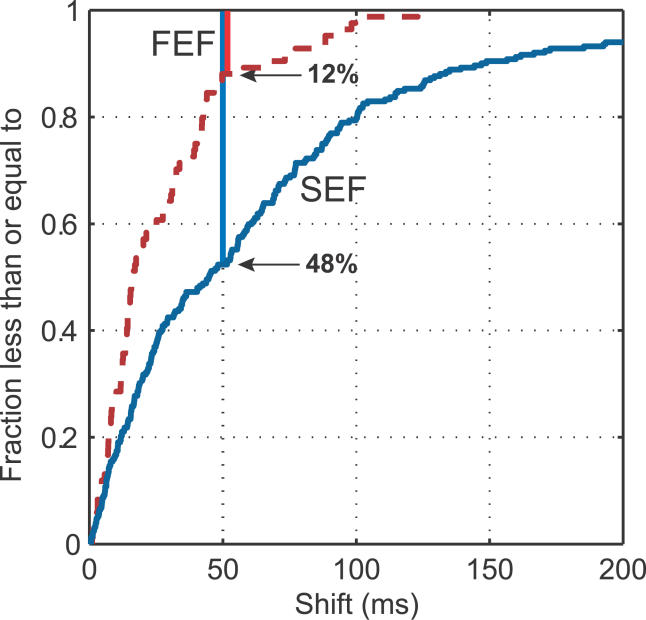
Comparison of FEF and SEF Shift Magnitudes Lines, cumulative distributions of shift magnitudes for FEF (dashed line) and SEF (solid line) sites. FEF effects are significantly weaker (Kolmogorov-Smirnov statistic, *p* > 10 ^−6^); e.g., only 12% of FEF shifts are greater than or equal to 50 ms, while 48% of SEF shifts are.

The SEF is heavily interconnected with the FEF, another frontal cortical area that is also critical for voluntary oculomotor control. So, we wondered whether similar results would be found with microstimulation there. Lesion or inactivation of the FEF leaves animals unable to produce oculomotor sequences from memory, while SEF lesions produce only subtle deficits [ 30, 31]. In contrast, a physiological study found more sequence-selective activity in SEF than FEF, suggesting that SEF plays a more significant role in oculomotor sequencing [ 24]. We found that microstimulation effects on oculomotor sequencing were much stronger in the SEF. Stimulation at 25 of 55 SEF sites (45%) produced a significant effect at one or more cue pairs ( *p* < 0.01, corrected for multiple comparisons), compared to only one FEF site (of 14; 7%). Figure 8 shows a cumulative distribution of the strength of behavioral effects following microstimulation in the FEF and SEF effects (as measured in milliseconds of shift, see above). All tested cue pairs are included and each data point is the SOA shift for that pair as a result of microstimulation (SEF: 55 stimulation sites with 286 sequence pairs; FEF: 14 stimulation sites with 84 pairs). Overall, the effects of microstimulation were much lower in the FEF than the SEF (Kolmogorov-Smirnov statistic: 0.36; *p* < 10 ^−6^).

## Discussion

These results demonstrate that microstimulation of the SEF during a memory delay affects the *order* of saccades to two remembered locations, but does not affect either the monkeys' ability to saccade to the locations that were cued or saccade parameters like accuracy, velocity, or latency. Indeed, stimulation seemed to affect the entire sequence rather than the individual movements. A bias of either the first or second saccade alone would have produced many target errors and divergent sequences. Instead, we found few target errors overall (and no increase with stimulation) and that stimulation seemed to bias the entire sequential movement so that its endpoint converged toward a contralateral zone.

Our results support hierarchical models of motor sequence control because they suggest that information about an action sequence can be dissociated from information about components of that sequence. This latter observation also may bring together two SEF results that have previously seemed unrelated: the evidence suggesting a role for the SEF in performance of oculomotor sequences [ 12, 13] and the fact that single contraversive, convergent saccades can be elicited by stimulation above oculomotor threshold [ 27, 28].

An important consideration in microstimulation studies is that while neurons near the electrode tip are activated by direct depolarization from the stimulation current [ 32, 33], neurons remote from the electrode tip are also likely to be indirectly activated [ 34– 36]. In fact, it is practically a certainty that when current is delivered to one cortical area to induce a behavioral change, there will be changes in activity in other areas of the brain as pathways are activated that underlie the alternative behavior caused by stimulation. So we also stimulated the FEF, an oculomotor cortical area to which the SEF is strongly connected. It was possible that our behavioral effects occurred via indirect activation of the FEF, but we instead observed much weaker sequence effects there. The stronger effects on sequencing that were triggered by stimulation of the SEF compared to the FEF makes it likely that the effects we observed are due to an influence on neurons in the SEF itself, or at least on circuits that run through the SEF and are engaged during normal brain function. Finally, another cortical area involved in oculomotor memory tasks that we did not examine is the lateral intraparietal area (LIP). We think it unlikely that this area is involved in sequencing, however, because delay-period memory activity in lateral intraparietal area has been previously shown [ 37] to reflect only the next upcoming movement and not to reflect other movements in a sequence.

In principle, microstimulation could have a net excitatory effect or inhibitory effect on cortical activity. Our results are consistent with excitatory effects. In our task, stimulation appears to exert its effect on the endpoint of the sequence by biasing sequence endpoints to converge to a point in contralateral space. This is highly consistent with results from previous work that employed single saccades and found excitatory effects of microstimulation. For example, Schiller and Tehovnik [ 29] applied subthreshold stimulation to the SEF at the time of the visual presentation of two cues and observed a contraversive bias in target selection. In addition, Newsome and colleagues stimulated the middle temporal area using trains of microstimulation similar in length and timing to ours [ 38, 39]. They observed a correspondence between neural activity and behavioral effects of microstimulation that only makes sense if the microstimulation had a net excitatory effect [ 40]. Finally, because our stimulation biased first saccades ipsiversively and second saccades contraversively, if there was any inhibition it must have acted on the first saccade. However, in that case, we might expect to observe divergence of saccade endpoints if a particular first saccade was selected by stimulation, where instead we saw convergence of saccade endpoints. These facts argue that our SEF stimulation had an excitatory effect on the sequence endpoint. Also, microstimulation did not seem to simply disrupt the monkeys' ability to discriminate target order or inject noise in a non-specific way. That would have produced a flattening of the slope of the psychometric function rather than the shift we observed. In sum, this suggests microstimulation had an excitatory effect that biased animals toward one possible saccade sequence for two given targets over the other, arguing for a specific role of the SEF in putting movements into a sequential order.

This is not to say that saccade sequencing is the only function of the SEF. SEF has been shown to be involved in a wide variety of different behaviors and to contain neurons that code for many different quantities, ranging from spatial variables like gaze endpoints to cognitive variables like conflict between several choices. However, several themes do emerge from these studies (for reviews, see [ 10, 28, 41]). First, it is clear that SEF is involved in coding for space, though the type of coding scheme that neurons use, whether head-, body-, or object-centered, seems to be variable. [ 42– 46]. Second, SEF neurons appear to be involved in tasks that require the association of a stimulus and response across time, whether this involves explicit memory or motor preparation (e.g., as first reported in [ 27]), or anticipation of a future response [ 47]. Third, the SEF seems to play a role in cognitive control and monitoring; it has been shown to signal the presence of conflict, behavioral errors, and reinforcement and to change as animals learn [ 48– 50]. In fact, our results are consistent with all three ideas, as our stimulation during a memory period appears to select a particular sequence in a top–down way without affecting the details of each individual movement.

It is also worth noting that our observations about oculomotor sequencing may generalize to sequencing of limb movements, as prior work has found both endpoint and sequence performance representations in cortical areas involved in limb movements. Dorsal premotor areas contain neurons that represent movement endpoints, while stimulation of ventral premotor cortex tends to elicit limb movements that converge toward a zone in contralateral space [ 51– 54]. Furthermore, a medial premotor area, the supplementary motor area, contains neurons selective for a given movement and its sequence position [ 55]. These premotor parallels to effects observed in SEF [ 12, 13, 27, 56] suggest that a corresponding representational scheme for sequence goals may also exist in the skeletal motor system. Coding of entire movement sequences in terms of their endpoints or goals in contralateral space may thus be a common mechanism by which action sequences are represented in the brain.

## Materials and Methods

### Subjects

We performed SEF stimulation experiments in two rhesus monkeys *(Macaca mulatta)*: monkey A (15 kg, male) and monkey B (5 kg, female). FEF stimulation was performed in monkey A. The animals were surgically implanted with a titanium head-holding device, and a recording chamber was placed stereotaxically over the medial frontal lobe and secured to the skull with cortical bone screws (Synthes, Chester, Pennsylvania, United States). Tungsten epoxy-coated electrodes (FHC, Bowdoinham, Maine, United States) were inserted transdurally for stimulation (electrode impedance poststimulation greater than or equal to 100 kOhm at 1 kHz). Surgeries were conducted under isoflurane anesthesia, and the animals were given analgesics as part of postoperative care. All animal procedures conformed to NIH guidelines and those of the MIT Institutional Animal Care and Use Committee.

### Behavioral task

The task is summarized in Figure 1. Fixation spots (0.2°) and cues (1.0–1.5°) were round white spots; the two cues always appeared at adjacent locations on a hexagonal grid (radius 5–13°). Seven SOA values were used. The SOA value, cue pair locations, and whether or not stimulation was delivered were all randomly intermixed from trial to trial. The time of appearance of visual stimuli was recorded with a photodiode and amplifier, and an LCD monitor was used (1850E, NEC America, Rancho Cordova, California, United States). Pixel rise time to half-maximum intensity was 6.1 ± 0.4 ms (mean + s.d.). Fixation was required within 1.0–1.75° of the fixation point; animals fixated within 0.5° on greater than 90% of trials. We analyzed eye movements during the delay; stimulation had no effect on the animals' fixational eye movements. To ensure that the animals made two separate movements, 350 ms of fixation was required after the first saccade; after the first saccade was completed, the fixation point appeared at the location of the first cue. The signal to begin each of the two saccades was the disappearance of the fixation point on which gaze was being held. The animals were rewarded for a sequence of saccades in the correct order. They were given a small reward (1/6 of the reward for a correct response) if the order was incorrect, to ensure continued task performance in the face of order errors. The animals chose incorrect locations on fewer than 2% of trials, regardless of stimulation; they received no reward in this case. For monkey A (19 sites) all possible cue locations were always indicated during the trial by a small (0.2°) dim spot at each location. For monkey B (36 sites), these were not present, the animal made saccades entirely from memory, and no difference in behavior was observed. We used all six possible pair locations in 39/55 sites, or 71% (36 of 36, animal B; 3 of 19, animal A). At other sites, a subset of the six was used to improve statistical power; once we determined that strong effects could be obtained using all six pairs at 50 μA or below, we used all pairs exclusively.

### Site localization

We placed the recording chambers stereotaxically over the frontal cortex between the superior branch of the arcuate sulcus and the midline. These brain landmarks were identified by structural MRI. Also, a reconstruction of the gray or white matter surface was computed from the MRI images and used to find the location of electrode penetrations, the recording chambers, and cortical sulci and gyri. For animal A, the highest-contrast boundary in the MRI images was between the gray matter and CSF, so we plotted the gray matter surface. For animal B, the highest-contrast boundary was between the gray and white matter, so we plotted the surface of the white matter.

To functionally localize the SEF, we mapped the cortex beneath the recording chamber by stimulating to elicit saccades, in a set of experiments prior to all the delay-period stimulation experiments reported here. For this suprathreshold stimulation, we used biphasic, cathodal-first square pulses, each phase 0.2 ms, in trains 200–400 ms long at 250–333 Hz. Initial stimulation was at 100–120 μA; once saccades were elicited, currents were reduced to determine thresholds. Saccades can be elicited from only a subset of SEF electrode penetrations at any depth (e.g., Schlag and Schlag-Rey [ 27] elicited saccades at only 66/121 electrode penetrations). Thus, to sample the SEF in an unbiased way, we did not restrict stimulation sites in this experiment to locations from which saccades could be evoked. During these experiments, we recorded signals from each electrode while it was being lowered into the brain, to determine the electrode location relative to the cell layer in cortex. When neural activity was seen, we stopped lowering the electrode and began the experiment.

Our stimulation sites were found 4–8 mm from the midline. While many investigators have found the SEF center between 2–5 mm from the midline (see, e.g., [ 27, 57]), there is variability in this location (for a review of the locations of lesion and physiology studies in SEF, see [ 28]). See, e.g., Figure 1 in Russo and Bruce [ 58], where in one hemisphere the SEF extends 3–5 mm laterally from the midline and in the other, 6–8 mm. Lateral to the SEF on the frontal lobe, some saccades can also be elicited from a subregion of dorsomedial premotor cortex (PMdr) at slightly higher thresholds [ 59]. Note, however, this subregion is separated from the SEF by a strip of cortex from which saccades cannot be evoked ([ 59], their Figure 1A). Given the variability previously reported in the location of the SEF, it is infeasible to completely rule out the possibility that a minority of the most lateral stimulation sites were outside this area. For the reasons above, however, it is probable that the large majority of our sites were in the SEF.

### Stimulation

On stimulation trials, stimulation was applied for the first 900 ms of the 1,000-ms delay period. These stimulation trains were sets of 0.2 ms per phase, biphasic, cathodal-first square constant-current pulses delivered at 250 Hz. We often used two stimulation electrodes (spaced at least 3 mm apart) for a single experiment; stimulation was delivered on separate trials to each electrode so that there could be no interaction between electrodes, and no dependence between effects of stimulation at the two electrodes was observed. If one stimulation electrode was used, stimulation was delivered on 33% of trials (67% unstimulated); if two were used, stimulation was delivered to each on 25% of trials (50% unstimulated). For animal A, 50 μA of current was used for each pulse phase unless that current elicited saccades during fixation, in which case the current was set below the threshold for eliciting saccades. For animal B, either 50 μA, 75 μA, or 120 μA of current was used unless similarly reduced due to elicited saccades. Forty-two of 55 sites were stimulated with less than or equal to 50 μA of current, of which 23 (42%) showed a significant effect. Within the SEF, we found no apparent topographical effects; e.g., there was no tendency for endpoints to be distributed above or below the horizontal meridian based on the site's position in the SEF.

The SEF is typically defined as the dorsomedial area from which saccades can be elicited at a threshold of less than or equal to 50 μA (i.e., where 50% of stimulation trains at less than or equal to 50 μA produces a saccade; [ 13, 27, 44, 60, 61], but cf. [ 62] at 100 μA). However, when animals are attempting to hold fixation, thresholds in the SEF increase dramatically [ 63]. In the work reported here, to determine saccade thresholds and map the SEF at a threshold of less than or equal to 50-μA, we applied stimulation either while animals scanned a computer monitor to detect a short flash or 200–300 ms after the end of a fixation period. Consistent with the prior results, if we then applied 50 μA of current during the delay period while animals were required to fixate, this stimulation did not elicit a saccade. The same range of current thresholds (less than or equal to 50 μA) was used to define the FEF [ 28, 64, 65], even though the average site in the FEF has a lower threshold than an average SEF site. For stimulation of FEF sites, we applied the same procedure to determine threshold, but since thresholds in the FEF increase by only a small amount during fixation, we reduced the current applied during the delay if stimulation elicited a saccade. In no case did stimulation elicit saccades on more than 10% of trials. The average current used in the FEF was 44.0 μA ( *N* = 14 sites, monkey A only). Thus, the currents used here are within the typical range used over many years of work on the frontal lobe eye movement areas. Finally, the number of pulses (stimulus train length) that we used was matched to the delay period, over which persistent memory activity was maintained. Our 900-ms trains are typical of other studies designed to affect behavior through subthreshold stimulation [ 38, 39].

The electrical stimulation passively spreads through the area at the tip of the electrode and activates a number of neurons: at 50 μA, high- and low-threshold units are activated over an estimated radius of, respectively, 0.1–0.5 mm [ 32, 66]. Thus, trains of microstimulation synchronize neurons in a small volume around the tip of the electrode, leading to stronger effects than if they each acted alone. So, the effect we observed may be one that relies on lateral interconnections (which connect neurons in this small volume, as they are concentrated isotropically near a given site in the cortex [ 67, 68]) as opposed to longer-range connections with other cortical areas.

### Data analysis

We used logistic regression to determine the size and significance of stimulation effects. This is a special case of a generalized linear model (GLM [ 69]) where the data are transformed by a logit link function ( Equation 1), and then a linear model is fitted using maximum likelihood. Using a probit link function produced qualitatively identical results. The model we fitted was:



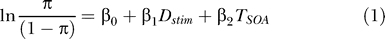



Here, β _0_ specifies the intercept (threshold), β _1_ specifies the slope of the psychometric function, and β _2_ gives the change in threshold (shift) due to stimulation. *D _stim_* is a dummy variable indicating the presence (1) or absence (0) of stimulation, and *T _SOA_* is the SOA time. Fitting the model specified the parameters β _0_, β _1_, and β _2_, and these were used to compute the best-fit curves, plotted as the solid lines in Figure 2. We also calculated 99% Wald confidence intervals around these best-fit curves [ 25]. Threshold-shift magnitude, in milliseconds, was computed as the difference between the medians (i.e., 50% points or LD50s) of the stimulated and unstimulated curves. Analysis was done with custom programs in MATLAB (MathWorks, Natick, Massachusetts, United States), Python ( http://www.python.org, and R ( http://www.r-project.org).

We report how stimulation consistently biased animals to more often saccade to the targets in a particular order. Another possible outcome was that stimulation would merely increase the animals' error rate without causing a consistent direction bias (i.e., “injected noise” into the order representation). This would be seen as a flattening in the psychometric curve around the 50% point (chance performance) in the stimulation case relative to the unstimulated case. To rule out this possibility, for every site and pair of cues in SEF we fit a logistic regression model that included an interaction term between the stimulation dummy variable and the SOA value. Only 6% of site and cue pair combinations (19/286) showed a significant effect of slope change in the absence of threshold shift at *p* < 0.05, almost exactly what would be expected by chance (not significantly different from 5%, *p* = 0.22, binomial test). Therefore, we rejected the hypothesis that stimulation flattened the curves around 50% and used the model shown above. For a more detailed discussion of these methods see [ 26].

It is also possible that performance on control (unstimulated) trials might change as a result of stimulation, e.g., as a result of increased likelihood of guessing a certain sequence when stimulation induced errors on that sequence. This was not observed, and we instead found that stimulation did not change the animals' performance on unstimulated trials. To show this, we computed the distribution of unstimulated curve thresholds (i.e., medians, LD50s, or 50% points) when there was a significant effect of stimulation and when there was no significant effect. These distributions were statistically identical ( *p* = 0.58, Kolmogorov-Smirnov test).

Because the animals' performance was so similar, we collapsed the data across animals as described above. To show quantitatively that the two animals' performance did not differ, we compared performance on control (unstimulated) trials and the number and size of shifts caused by stimulation across animals. First, since animals were trained until their performance on the task was high, control (i.e., unstimulated) performance was not significantly different, with median unstimulated biases (absolute values of the threshold) of 34 ms and 44 ms for animals A and B, respectively (n.s., *p* = 0.07, Wilcoxon test). Second, the effect of stimulation was identical across animals. Stimulation produced a significant shift in 69/192 (36%) pairs for animal A and 25/60 (42%) for animal B; not significantly different ( *p* = 0.57, Fisher exact test). Third, the median effect size, computed over all stimulated sites, was also statistically the same ( *p* = 0.38, Wilcoxon rank-sum test): 30 ms for animal A and 45 ms for animal B. Since the effects (on stimulation especially) were so similar across animals, we pooled their data for analysis purposes; furthermore, the effects that we observed in the pooled data and that we report here were also seen in each animal's data individually.

To determine the number of stimulation sites that showed a significant effect, we tested if any of the pairs used at that site showed a significant threshold shift at *p* < 0.05, using the Bonferroni correction for multiple comparisons.

Figure 7A, 7B, and 7D are calculated using endpoint difference vectors. These were found for each significant ( *p* < 0.05) cue pair by subtracting the vector from the fixation point to the endpoint selected by stimulation and the vector of the non-selected endpoint. We considered only vectors at sites with more than one significant pair for all analyses on difference vectors, as a mean could only be calculated with two or more difference vectors. For each site, we calculated the mean of the difference vectors for those pairs (the “convergence zone”) and rotated the vectors such that the mean vector pointed in the 0° direction. To determine whether the observed concentration about the mean was due to chance, we calculated the null distribution of a set of endpoint vectors about their mean, matched to our dataset. First, we simulated the null distribution of difference vector directions, for that number of pairs, using the same target locations used in the experiment. The simulation produced the null probability distribution of the difference vectors when the identity of the significant pairs and their shift directions were chosen randomly. We then averaged these null distributions, matched to each site, over all sites. We plotted all vectors by aligning all of their endpoints at the origin in Figure 7A; to evaluate the significance of the effect shown there, we computed the expected number (by chance) of vectors whose angles were more than 90° away from the site's mean, using the null distribution described above.

In order to plot the observed and simulated distributions with respect to a scalar quantity, we calculated the absolute difference, in degrees, of each difference vector and the mean for that site. The corresponding observed and null distributions are shown in Figure 7B. A two-sample Kolmogorov-Smirnov test was used to determine the significance of the difference between the two distributions.

## Abbreviations

FEF: frontal eye field
SEF: supplementary eye field
SOA: stimulus onset asynchrony
